# Music training and language learning improve verbal memory performance but do not change white matter characteristics of the splenium: a longitudinal DTI study

**DOI:** 10.3389/fpsyg.2025.1659705

**Published:** 2025-10-17

**Authors:** Anja-Xiaoxing Cui, Yujin Choi, Negin Motamed Yeganeh, Nancy Hermiston, Janet F. Werker, Lara A. Boyd

**Affiliations:** ^1^Space for Interdisciplinary Experiments on Sound, Department of Musicology, University of Vienna, Vienna, Austria; ^2^Vienna Cognitive Science Hub, University of Vienna, Vienna, Austria; ^3^School of Music, University of British Columbia, Vancouver, BC, Canada; ^4^Department of Psychology, University of British Columbia, Vancouver, BC, Canada; ^5^Brain Behaviour Laboratory, Faculty of Medicine, University of British Columbia, Vancouver, BC, Canada

**Keywords:** music training, language learning, verbal memory, corpus callosum, splenium, white matter characteristics, diffusion tensor imaging

## Abstract

**Introduction:**

Previous research has suggested associations between memory performance, white matter characteristics, and training in music performance. Associations of memory performance and white matter characteristics have also been found with language learning. Given the proposed links between music training and language learning, we investigate here, whether a year of different types of training (music, language, both, or other), related to white matter characteristics in the corpus callosum and the fornix and verbal and visuospatial memory performance changes.

**Methods:**

We obtained verbal and visuospatial memory performance scores (California Verbal Learning Test Second Edition; Wechsler Memory Scale Fourth Edition, Designs 1 and 2) and diffusion tensor imaging data from 65 young adult participants before and after a year during which they enrolled in music performance classes, language learning classes, both types of classes, or other types of classes.

**Results:**

Analyses revealed a significant linear contrast of class type showing improvements in verbal memory scores for participants who had taken either music performance training or language learning classes, and the biggest improvement for participants that had taken both types of classes. No significant effects were observed for visuospatial memory. Regression analyses further indicated that fractional anisotropy in the splenium at time point 1 significantly predicted verbal memory change but that the class type factor did not add explanatory power.

**Discussion:**

Our findings indicate that both music training and language learning can lead to verbal memory improvements and that both types of training can compound, for example, in the form of singing or opera training to lead to greater improvements. Thus, training in music performance and language learning may have additive effects on verbal memory improvements. While callosal white matter characteristics related to verbal memory changes in our sample, the neural mechanism of the shown training effects is presumably another.

## 1 Introduction

### 1.1 Music training and memory

Several studies have reported that people with musical experience outperform people without musical experience in memory tests, suggesting possible effects of music training on memory (e.g., Talamini et al., 2017). A meta analysis on 29 studies published between 1987 and 2017 concluded that musicians have better memory than non-musicians based on their performance in long-term, short-term, and working memory tests (Talamini et al., 2017). The findings indicated advantages for musicians specifically in verbal memory tasks, but no significant advantage was found for visuospatial stimuli. Findings from longitudinal studies on children support the conclusions of the meta-analysis (Ho et al., 2003; Rickard et al., 2010; Roden et al., 2012). In a more recent study on adult participants with or without mild traumatic brain injury, eight weeks of music training significantly improved performance on the California Verbal Learning Test (CVLT) - a test of verbal memory - for both groups (Vik et al., 2018). Greater performance on the CVLT has also been found in a recent cross-sectional study of older adult musicians compared to older adult non-musicians (Galloway and Strong, 2021). In another report, the number of years of music training and the current hours playing an instrument per week of 58 older adults was significantly associated with CVLT scores (Strong, 2022). The overall scarcity of longitudinal studies with the exceptions summarized above however, precludes conclusions about the causal effects of music training on memory.

### 1.2 Language learning and memory

As language acquisition and processing is dependent on working memory (Szmalec et al., 2012), an association between language learning - or the desired outcome of language learning, that is, bilingualism or multilingualism - and memory may be hypothesized. To this effect, a meta-analysis on working memory capacity reported that bilinguals had greater working memory capacity compared to monolinguals (Grundy and Timmer, 2017). The meta-analysis studied effect sizes from 27 studies from 1995 to 2015. Since 2017, data from other studies has further supported the idea of a memory advantage through having learning multiple languages, both in young adults (Cockcroft et al., 2019) and a sample of highly educated older adults (Fyndanis et al., 2023).

There are few longitudinal studies examining the relationship between multilingualism and memory. In one of the few, an association between memory and language learning outcomes was found, though memory was not tested before and after language learning (Kormos and Sáfár, 2008). The direction of causality therefore remains unclear despite the longitudinal design. On the other hand, in a study of healthy older adults, aged 65 to 75, an 11-weeks long program of foreign language training did not lead to improvements in associative memory (Berggren et al., 2020). These competing results suggest that the effects of language learning on memory may depend on the age at which the additional languages are learned, as well as the method, with which memory is tested.

### 1.3 Memory and the brain

Brain plasticity related to memory performance has been the focus of numerous studies (e.g., Charlton et al., 2010). For example, a longitudinal study exploring changes over two years in white matter indicated that changes in white matter coincided with changes in cognitive performance in healthy adults (Bender et al., 2016). Despite the evident importance of white matter structure to cognitive faculties, studies on the relationship between white matter and memory remain scarce relative to studies on the relationship between grey matter and memory (Filley and Fields, 2016; Golestani et al., 2014; Lazar, 2017). Therefore, we focus here on the white matter characteristics of the human brain related to memory by studying measures obtained through diffusion tensor imaging (DTI). DTI is a magnetic resonance imaging technique enabling the approximate visualization and characterization of white matter tracts by capitalizing on differences in the diffusion characteristics of water in the presence of myelin (Assaf and Pasternak, 2008). The effects of development on white matter tracts is profound (Lebel et al., 2019) and likely overshadows the impact of additional training. Given that our study focuses on adult brain characteristics, we do not include references to DTI studies from child or adolescent participants in this introduction though they exist (Habibi et al., 2018; Loui et al., 2019; Mohades et al., 2015).

One review highlights the role of white matter tracts as part of the neural networks which are activated during working memory tasks (Lazar, 2017). Several papers have reported associations between working memory and white matter (Davis et al., 2012; Golestani et al., 2014; Tamnes et al., 2011), although DTI studies on young adults were relatively rare compared to those with other age groups (Lazar, 2017), perhaps due to the particular relevance of neuroplasticity during early development and its opposite, neurodegeneration during later adulthood, emphasizing that white matter provides the myelinated systems that support the structural connections of neural networks for cognitive functions.

Some studies have explored which white matter tracts are of particular relevance to memory functions (e.g., Kennedy and Raz, 2009). One cross-sectional study, demonstrated that white matter characteristics in the prefrontal cortex and the genu of the corpus callosum (CC), the white matter tract connecting the two hemispheres, are linked to working memory performance (Kennedy and Raz, 2009). Similarly, in a study of 40 young adults, a positive relationship between fractional anisotropy (FA), a measure of white matter integrity, obtained by measuring how directional the diffusion of water molecules is within a given voxel or region (though there is debate about the interpretation of different DTI-based measures, see Figley et al. (2022)), in the genu of the CC and memory was suggested (Tang et al., 2010), thus suggesting that higher white matter integrity in the CC is related to memory performance. A possible relationship between CC characteristics and memory has also been supported by evidence from patients with traumatic brain injury as well as agenesis of the CC (Arenth et al., 2014; Erickson et al., 2014) whose performance on the CVLT is worse than that of healthy control participants, indicating a link between CC integrity and memory performance.

Longitudinal evidence also points to an important role of the fornix, the major output white matter tract of the hippocampus, for memory; fornix volume and axial diffusivity, another measure of white matter integrity, were predictive of cognitive decline in healthy older adults (Fletcher et al., 2013). A systematic review of 143 studies on the relationship between episodic memory scores and white matter characteristics of the fornix indicated a robust relationship between these two variables throughout development and in several patient groups (Douet and Chang, 2015). Overall, these findings highlight the potential role of white matter characteristics of the fornix and the CC in memory function across the lifespan, although further longitudinal studies are needed to investigate causal relationships, particularly in young adulthood.

### 1.4 Music training and the brain

Several studies have also utilized DTI to explore possible CC and fornix differences between musicians and non-musicians. In a review of such studies, researchers have concluded that differences have been frequently reported for the CC, more so than differences in intra-hemispheric fibres, the internal capsule or the corticospinal tract (Moore et al., 2014). More recent reports support this conclusion. For example, in one study comparing professionally trained instrumentalists and non-musicians, differences were found in the CC, such that musicians had lower FA values in this cross-hemispheric tract (Acer et al., 2018). In a sample of absolute pitch and non-absolute pitch musicians, the age of onset of musical training was negatively correlated with FA in the white matter pathway connecting the left and right planum temporale, which is localized to the splenium of the CC, suggesting experience-dependent effects to the CC (Leipold et al., 2021). In another cross-sectional study, integrity of the posterior CC was related to choir singing experience (Moisseinen et al., 2024). Additionally, older adults exhibited specific positive effects of choir singing on the bilateral fornix, a change not seen in other age groups.

However, not all findings align with this conclusion. A cross-sectional study reported that musicians had significantly lower FA values in both the left and the right corticospinal tracts than non-musicians, but no significant group differences in other areas including the CC (Imfeld et al., 2009). Others have found white matter characteristic differences in the splenium of the CC between musicians and non-musicians using mean diffusivity but not FA as a measure (Leipold et al., 2021). Further, the direction of the effect is unclear, as others have also reported greater FA in the genu of the CC in participants who had received continuous music training during childhood and adolescence compared to participants who had not received such training (Schmithorst and Wilke, 2002). Similarly, greater FA in the CC has also been reported for early-trained compared to late-trained musicians matched for years of training and experience (Steele et al., 2013). Recent findings also suggest, that some of the effects may be limited to the specific type of music training received, where voxel-based morphometry differences in the CC and the fornix were only related to the training received on the piano but not on wind instruments (Choi et al., 2021).

Nevertheless, evidence from one longitudinal study on musically naive healthy older adults further suggests a particular effect of music training to the fornix (Jünemann et al., 2022). Those who participated in a music listening and music theory program showed a decline in fornix fiber density, as opposed to participants who had received six months of piano training. Changes in fornix fiber density was positively associated with the amount of weekly piano practice.

### 1.5 Language learning and the brain

In contrast to studies suggesting an effect of music training on both the CC and the fornix, there are more studies suggesting effects of second language learning on the CC than on the fornix. Differences in the fornix have been reported by Gold et al. (2013) and Midrigan-Ciochina et al. (2024). The evidence pointing toward an effect of second language learning on the CC is more robust (Coggins et al., 2004). A recent meta-analysis examining 23 studies published between 2011 and 2022 on white-matter correlates of bilingualism concludes that studies have reliably shown differences in white matter characteristics for bilingual compared to monolingual individuals in a number of white matter tracts such as the left superior longitudinal fasciculus, and the forceps major and minor of the CC (Anderson et al., 2024).

The direction of the effect however is unclear. While some have reported lower FA values in bilingual compared to monolingual individuals’ CC (Kuhl et al., 2016; Mohades et al., 2012; Anderson et al., 2018; Midrigan-Ciochina et al., 2024), others have reported higher FA values (Luk et al., 2011; Pliatsikas et al., 2015) and a negative relationship between FA values and onset of second language learning (Luk et al., 2020). High levels of language training, as well as the number of languages spoken, were also associated with lower FA in the genu and the splenium of the CC in a study comparing professional simultaneous interpreters to control participants (Elmer et al., 2011). On the other hand, others have reported a positive association between FA in the anterior midbody of the CC with the age of acquisition for a second language as well as higher FA in tracts including the forceps minor of the CC being associated with greater proficiency (Nichols and Joanisse, 2016).

Longitudinal studies further add to the conflicting evidence regarding a link between second language learning and changes in white-matter characteristics. In one longitudinal study, white matter changes were compared between participants enrolled in a foreign language class and control participants (Schlegel et al., 2012). In summary, research on the link between multilingualism and changes in the CC and fornix present mixed evidence, with more studies reporting possible effects on the CC than on the fornix but unclear direction of association between additional language experience and FA in the CC.

### 1.6 Relationships between music training, multilingualism, memory, and the brain

In the previous sections, we summarized relevant, existing evidence regarding the links between two variables at a time: 1.1 music training and memory, 1.2 multilingualism and memory, 1.3 memory and the brain, 1.4 music training and the brain, and 1.5 multilingualism and the brain. The evidence regarding a connection between music and language in general, and the possibility of connections between music training and language learning, has been discussed at length elsewhere (Besson and Schön, 2001; Jäncke, 2012; Mcmullen and Saffran, 2004; Moreno, 2009; Nayak et al., 2022; Patel, 2003; Slevc, 2012). Only very few studies have explored how three of the four variables may interrelate. We summarize the evidence from studies on 1.6.1 music training, memory, and the brain, 1.6.2 language learning, memory, and the brain, 1.6.3 music training, language learning, and the brain, and 1.6.4 music training, language learning, and memory below.

#### 1.6.1 Music training, memory, and the brain

The relationship between music training, memory, and underlying white matter characteristics has been discussed in a few studies. Although there are no clear, consistent relationships, DTI studies have provided evidence supporting a role of the fornix or CC in possibly explaining the memory advantage in musically trained participants. In a cross-sectional DTI study comparing 18 musicians, 19 dancers, and 19 control participants, the musicians outperformed both other groups on a working memory task (Giacosa et al., 2016). Analysis of the white-matter tracts of participants revealed that musicians and dancers showed differences in white-matter characteristics of the CC. In a longitudinal study on older adults, the relationship between changes in the white-matter characteristics of the fornix and changes in episodic memory abilities, though small to moderate, was significant (Jünemann et al., 2022). The neural correlate of a possible” musician’s memory benefit” thus remains unclear.

#### 1.6.2 Language learning, memory, and the brain

Given the scarcity of longitudinal evidence, a more precise wording for the title of this section would be the relationship between multilingualism, memory, and the brain. In a cross-sectional study comparing 40 cognitively healthy older adult bilinguals with 38 cognitively healthy older adult monolinguals, the monolingual participants had poorer outcomes on a test which included a measurement of verbal memory than the bilingual participants (Berkes et al., 2021). The memory advantage for the bilingual participants was accompanied by lower FA scores in these participants though the neuropsychological and neuroimaging measures were not directly related to each other in the study. Further, the researchers did not specify which brain structures may drive the effect. In another cross-sectional study on 82 participants, half of whom were considered bilingual, analyses revealed a significant correlation between memory performance and FA in the splenium of the CC across the whole sample and within the bilingual group, but not within the monolingual group (Macbeth et al., 2021).

The white matter characteristics underlying the bilingual advantage in memory has also been studied in 88 participants with mild cognitive impairment. Of those, 35 were” active” bilinguals who could both speak and understand Spanish and Catalan. The remaining 53 Spanish speakers were considered a” passive” bilingual group as they could understand but could not speak Catalan (Marin-Marin et al., 2020). All participants were older adults (mean age = 73.10) and had mild cognitive impairments. No significant differences were observed in their performance on neuropsychological tests, however, DTI analyses revealed that active bilinguals exhibited significantly higher mean diffusivity, indicative of lower white matter integrity, in the fornix than passive bilinguals.

#### 1.6.3 Music training, language learning, and the brain

To the best of our knowledge, no study has directly related music training, language learning, and the white matter characteristics of the regions-of-interest which we have outlined above, namely, the CC and the fornix. Others have contrasted groups of musicians and bilinguals in their brain activity using functional magnetic resonance imaging (Elmer and Jäncke, 2018; Musacchia et al., 2007) or electroencephalographic measures (Besson and Schön, 2001; François and Schön, 2011). These studies suggest that both types of training modify brain activity though not always in a comparable manner (Johansson, 2008; Zatorre, 2013). Where both music training and language learning were related to white matter characteristics, the research focus was another such that the ROI was the arcuate fasciculus or other white matter tracts apart from the fornix or the CC (Cui et al., 2024; Sihvonen et al., 2021; Vaquero et al., 2020).

In one study relating music training, white matter characteristics of the CC, and a languagerelated variable, the latter was phonetic processing: White matter characteristics of the CC differed between professional musicians and non-musicians and these characteristics were significantly related to their performance in a phonetic categorization task (Elmer et al., 2016).

#### 1.6.4 Music training, language learning, and memory

Studies on music training, language learning and memory are even more scarce. One study explored differences in working memory abilities between 14 control participants (monolingual nonmusicians), 14 monolingual musicians, and 13 bilingual non-musicians (Alain et al., 2018). Musicians significantly outperformed control participants and bilinguals in a sound identity working memory tasks. A non-significant group advantage for musicians and bilinguals over control participants was found for a sound location working memory task. In a sample of 82 academics, the number of known foreign languages significantly correlated with verbal memory though the number of hours per week currently playing instruments did not (Fyndanis et al., 2023). Results from studies on 153 young adults belonging either to a group of monolingual musicians, bilingual musicians, monolingual non-musicians, and bilingual non-musicians (D’Souza et al., 2018; Moradzadeh et al., 2015) suggest enhanced working memory through music training but not language learning.

### 1.7 Music training, language learning, memory and the brain

Although there are a number of studies about the correlations between some of our variables of interest, there is a significant lack of research on how all four variables relate to each other. Our study aims to start filling this gap. Sixty-five young adult participants, recruited from different programs at a selective university, including programs focused on music training and/or language learning, were assessed in their verbal and visuospatial memory before and after one year of education. FA of the CC and fornix were also measured at these time points. In our study, we thus ask specifically, is there a difference in the change of verbal and visuospatial memory performance between participants receiving different kinds of training? Based on the available evidence, we hypothesized that participants receiving music and/or language training would show improvements in verbal memory performance. We also asked, does this improvement relate to white-matter characteristics of the CC and fornix? Based on the available evidence, we hypothesized that improvements in verbal memory performance would relate to FA measures of the CC and fornix.

## 2 Methods

The studies involving humans were approved by the University of British Columbia Office of Research Ethics. The studies were conducted in accordance with the local legislation and institutional requirements. The participants provided their written informed consent to participate in this study.

### 2.1 Participants

Data were collected from 65 young adults (forty-seven identified as women, mean age: 21.3, SD: 3.4, at time point 1) after obtaining informed consent. Data were acquired at two time points, spaced one year apart. During the intervening year, participants engaged in different types of training through their enrollment in different classes at a university with competitive admission requirements, such as music, language, and other classes. Participants were separated into four groups: Those who were enrolled in both language learning and music performance classes (*n* = 14), language learning classes but no music performance classes (*n* = 23), music performance but no language learning classes (*n* = 14), and those who were enrolled in neither (*n* = 14). Subsequently for simplicity’s sake, we refer to the group enrolled in language and music performance classes as” Both,” the group only enrolled in language classes as” Language,” the group only enrolled in music classes as” Music,” and the group not enrolled in either type of classes as” None.” ANOVAs indicated that groups differed in their previous musical experience, *F*(3,62) = 20.11, *p* < 0.001, η^2^_p_ = 0.49, and language experience, *F*(3,60) = 11.00, *p* < 0.001, η^2^_p_ = 0.36, as measured using the training subscale of the Goldsmiths Musical Sophistication Index (GOLD-MSI, Müllensiefen et al., 2014) and a multilingual proficiency index calculated from participants’ self-assessment on the Language Experience and Proficiency Questionnaire (Kaushanskaya et al., 2020, see Cui et al., 2024 regarding details on how the index was calculated), see Tables 1, 2 for *post hoc* tests. GOLD-MSI is a scale developed to assess different music-related expertise. A greater value on the training subscale indicates a greater amount of past formal music training, and a higher multilingual proficiency index indicates that the participant has greater multilingual proficiency.

**Table 1:** Table shows group differences in musical sophistication as indexed through the GOLD-MSI.

Group 1	Group 2	Mean Differences	SE	*t*	Cohen’s *d*	*p*_Tukey_
None	Language	−0.95	2.66	−0.36	−0.12	0.984
None	Music	−16.00	2.99	−5.35	−2.02	<0.001
None	Both	−15.79	2.99	−5.28	−1.99	<0.001
Music	Language	15.05	2.66	5.66	1.90	<0.001
Music	Both	0.21	2.99	0.07	0.03	1.000
Language	Both	−14.84	2.66	−5.57	−1.88	<0.001

*Post hoc* tests revealed significant differences between groups that were enrolled in music performance classes (music, both) and those that were not (language, none). Notably, there was no significant difference between the music and both groups.

**Table 2:** Table shows group differences in multilingual proficiency as indexed through the LEAP-Q.

Group 1	Group 2	Mean differences	SE	*t*	Cohen’s *d*	*p*_Tukey_
None	Music	−0.13	0.15	−0.86	−0.33	0.825
None	Language	−0.59	0.14	−4.36	−1.50	<0.001
None	Both	−0.70	0.15	−4.51	−1.77	<0.001
Language	Music	0.46	0.13	3.48	1.17	0.005
Language	Both	−0.11	0.14	−0.78	−0.27	0.862
Music	Both	−0.57	0.15	−3.74	−1.44	0.002

*Post hoc* tests revealed significant differences between groups that were enrolled in language learning classes (language, both) and those that were not (music, none). No significant difference was found between language and both groups.

### 2.2 Data acquisition

#### 2.2.1 Neuroimaging

From each participant, we obtained a high-resolution T1 scan (TR = 7.4 ms, TE = 3.7 ms, flip angle *θ* = 6°, FOV = 256 mm, 160 slices, 1 mm thickness, scan time = 3.2 min). Furthermore, we obtained diffusion weighted scans using a high-angular resolution diffusion imaging sequence across 60 non-collinear diffusion gradients (b-value = 700 s/mm2, TR/TE = 7015/60 ms, voxel size = 2 × 2 mm, FOV = 224 × 224 × 154 mm, slice thickness = 2.2 mm) and three T2 scans with a b-value of *θ* (TR = 5,266 ms, TE = 68 ms, acquired voxel size = 2.2 mm × 2.2 mm × 2.2 mm reconstructed in plane to 2 mm with *θ* mm slice gap, FOV = 224 × 226 mm, scan time = 5.5 min).

#### 2.2.2 Neuropsychology

Participants’ verbal learning and verbal memory ability were assessed using the California Verbal Learning Test Second Edition (CVLT-II) (Delis et al., 2000). The test comprises multiple trials and includes different subscales. For example, it assesses participants’ free or cued recall after a short or long delay. The CVLT-II has high test–retest reliability in healthy adults and its usefulness in longitudinal study designs has been demonstrated before (Woods et al. (2006)). To assess visuospatial memory, we used the Designs subtest of the Wechsler Memory Scale - Fourth Edition (WMS-IV) Wechsler Memory Scale (WMS) (Wechsler, 2009; Carlozzi et al., 2013). Participants were asked to perform various tasks, such as drawing or selecting designs. CVLT-II and WMS-IV were administered by a trained neuropsychologist.

### 2.3 Data pre-processing

#### 2.3.1 Neuropsychology

Subscale values were calculated and converted to percentile scores according to the test manual. To avoid weakening our statistical power for our later analyses, we sought to determine which subscale could be used as a representative measure of verbal learning and memory. We calculated Pearson correlation indices between all subscale values at time point 1. These analyses revealed that the CVLT-II short delay free recall subscale had the highest average correlation with every other subscale. We therefore selected this subscale as the most representative. The same analyses on the subscale values at time point 1 of the Designs subtest of the WMS-IV revealed that Design 1 total was the most representative subscale. The chosen subscales for verbal memory and visuospatial memory were not significantly correlated (*p* = 0.121).

Correlation analyses were conducted to check whether changes in these selected subscale measures of verbal or visuospatial memory were related to the participants’ previous experience in music or language, as indicated through the GOLD-MSI and the multilingual proficiency index, revealing no significant effects (*p*s *> 0*.05).

#### 2.3.2 Neuroimaging

Data preprocessing was performed with FMRIB’s FSL suite version 5.0.91 (Smith et al., 2004). The preprocessing pipeline included the following steps: Eddy current and head motion correction was done by a 3D affine registration (Jenkinson and Smith, 2001). The b-vectors gradient matrix was rotated by these tools as well. The brain extraction tool was used to extract the brain (Smith, 2002) before eigenvectors of diffusion tensors were calculated for each voxel and FA values using the dtifit function in FSL. Then, the bedpostx function was employed to calculate the probability distribution of fiber directions for each voxel (Behrens et al., 2007). A mask for the fornix was created based on the Fornix-FMRIB-FA1mm Template (Brown et al., 2017). The JHU DTI-based white-matter atlas was used to create masks for the genu, body, and splenium of the CC (Mori et al., 2005; Wakana et al., 2007; Hua et al., 2008). Masks were created in MNI space (1 mm), then co-registered to each participant’s T1 space (1 mm). Afterwards, masks were co-registered to diffusion space for each participant, then thresholded at 50% and binarized. Next, we extracted for each participant the average FA value within their fornix and their CC.

### 2.4 Statistical analysis

Statistical analysis was done in JASP (JASP Team, 2025). All our analyses included age and sex covariates given their likely influence on white matter characteristics (e.g., Shin et al., 2005; Kochunov et al., 2011).

#### 2.4.1 Effects of different training types on memory changes

We first employed two repeated measures ANCOVAs to investigate the effects of type of experience on changes in verbal and visuospatial memory performance each. In the first analysis, we investigated the effect of music training, that is, we grouped participants together who were enrolled in music classes and compared the change in their performance to those of participants who had not enrolled in music classes. In the second analysis, we investigated the effect of language learning on changes in verbal and visuospatial memory, grouping together participants who were enrolled in language classes and comparing changes in their performance to those of participants who had not enrolled in language classes. As we were specifically interested in possible changes in the music training and language learning groups respectively, we followed up each ANCOVA with *post hoc* tests on the effect of time in the music training group and the language learning group, respectively. An ANCOVA with a linear contrast was next used to follow-up results from the repeated measures ANCOVAs to explore the impact of the combination of musical training and language learning on changes in verbal memory.

#### 2.4.2 Correlation between white matter characteristics and verbal memory changes

Correlation analyses were then performed to examine whether changes in verbal memory related to FA in the genu, body, and splenium of the CC and the fornix at time points 1 and 2. Next, we conducted linear regressions to assess the influence of FA values of the splenium at time point 1 on verbal memory change, and the influence of verbal memory change on the FA values of the splenium at time point 2. To examine the effect of experience, we included group as a factor. Lastly, a repeated measures ANCOVA was conducted to examine the effects of group, time, on FA values in the splenium, to determine whether different types of training influence FA values in the splenium across time.

## 3 Results

### 3.1 Memory performance

#### 3.1.1 Verbal memory test comparisons

**Figure 1:**
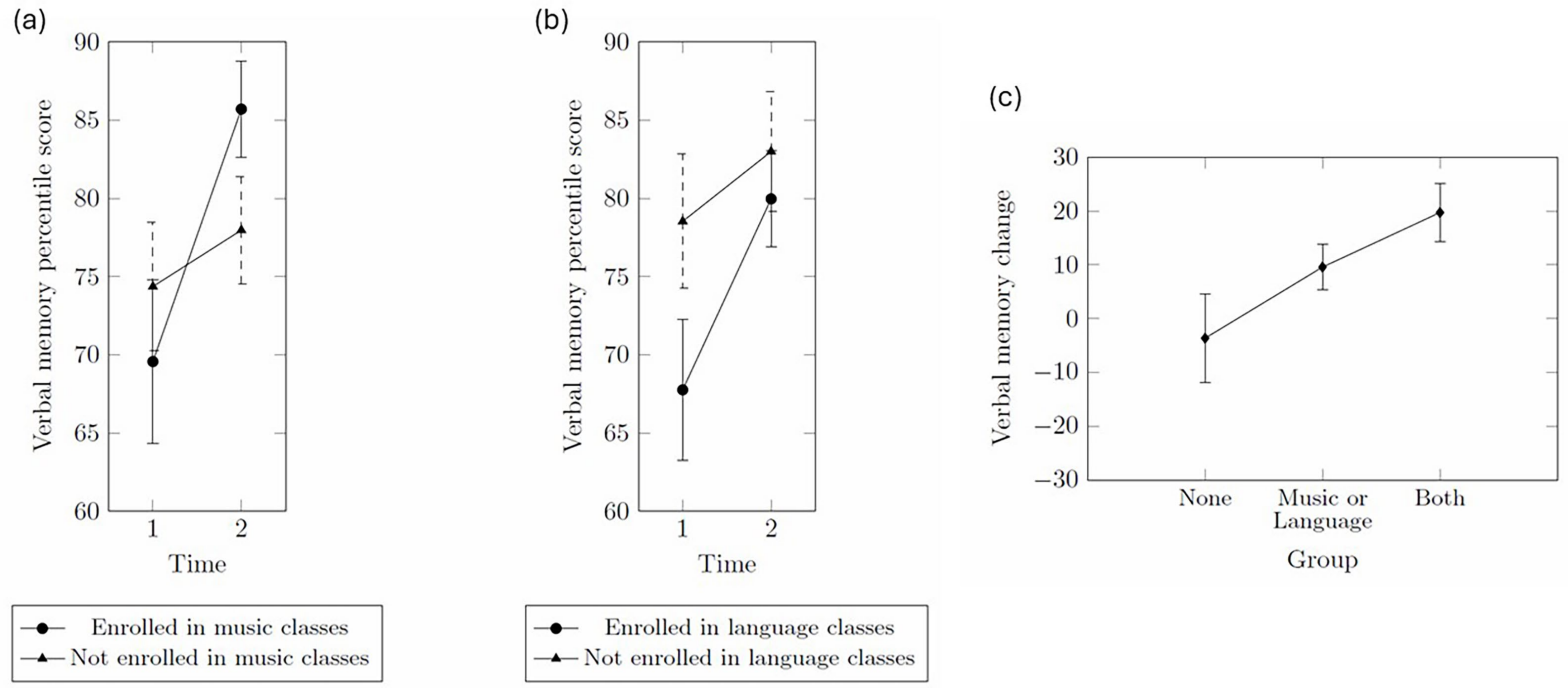
**(a)** Verbal memory percentile scores over time in the participants who are enrolled in music classes and those who are not. **(b)** Verbal memory percentile scores over time in the participants who are enrolled in language classes and those who are not. **(c)** Comparison of verbal memory changes over time between groups. The ‘None’ group refers to participants who did not enroll in either music or language classes. The ‘Music or Language’ group includes those who enrolled in either music or language classes. The ‘Both’ group refers to participants who enrolled in both music and language classes.

The repeated measures ANCOVA checking for the effect of music training on verbal memory revealed no significant main or interaction effects, *p*s *> 0*.05 (Figure 1a). Planned *post hoc* tests, Tukey corrected for multiple comparisons, revealed that there was a significant increase, *p* = 0.019, Cohen’s *d* = 0.70, in verbal memory scores within the music training group between the two time points. In contrast, participants who had not received music training did not score higher at time point 2 compared to time point 1. No significant differences were found for the between group comparisons, *p > 0*.05.

Next, the repeated measures ANCOVA checking for the effect of language learning on verbal memory revealed no significant main or interaction effects, *p*s *> 0*.05 (Figure 1b). Planned post hoc tests, Tukey corrected for multiple comparisons, revealed that there was a significant increase, *p* = 0.019, Cohen’s *d* = 0.49, in verbal memory percentile scores within the language learning group between the two time points. In contrast, participants who were not enrolled in language learning classes did not score higher at time point 2 compared to time point 1. No significant differences were found for the between group comparisons, *p > 0*.05.

The follow-up ANCOVA to clarify the group effect revealed a significant linear contrast on verbal memory change, such that None *<* Music or Language *<* Both, *p* = 0.036, *d*_w_ = 0.60 (Abelson and Prentice, 1997), as shown in Figure 1c.

#### 3.1.2 Visuospatial memory test comparisons

**Figure 2:**
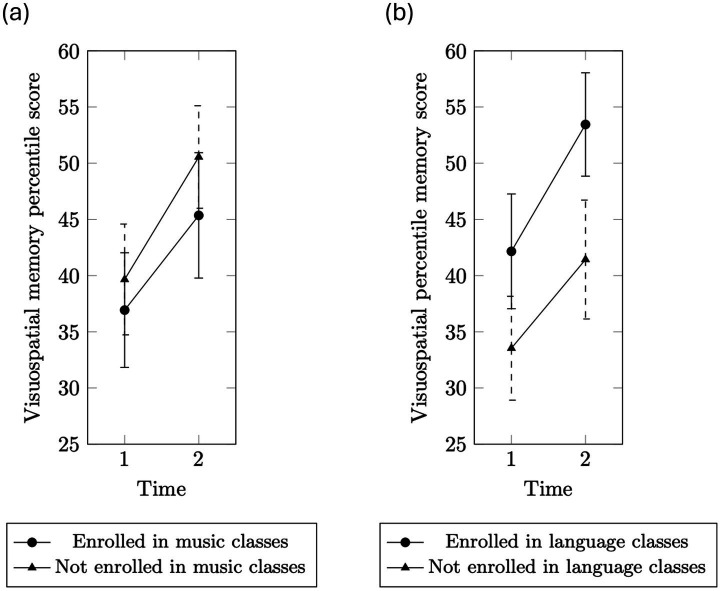
**(a)** Visuospatial memory percentile scores over time in the participants those are enrolled in music classes and those are not. **(b)** Visuospatial memory percentile scores over time in the participants those are enrolled in language classes and those are not.

The same analyses were conducted to examine visuospatial memory changes across groups. No significant effects were found in the repeated measures ANCOVAs and post hoc tests (Figures 2a,b). Accordingly, no follow-up ANCOVA was conducted. Our next analyses thus focus exclusively on the relationship between verbal memory performance and white matter characteristics.

### 3.2 White matter characteristics

Based on the results of the previous analysis, we examined the relationship between FA values in our ROIs and verbal memory change, at both time points, finding significant positive correlations between FA values in the splenium of the CC and verbal memory change at both T1, *r*(63) = 0.33, *p* = 0.008, and T2, *r*(63) = 0.32, *p* = 0.009 (Table 3).

**Table 3:** Correlation coefficients between verbal memory change and FA values in the ROIs.

Time	ROI	Person’s *r* (*p*-value)
T1	Body of the CC	0.12 (0.345)
T2		0.11 (0.378)
T1	Fornix	−0.15 (0.228)
T2		−0.11 (0.37)
T1	Genu of the CC	−0.13 (0.287)
T2		−0.11 (0.398)
T1	Splenium of the CC	0.33 (0.008)*
T2		0.32 (0.009)*

Next, we conducted a linear regression analysis to examine whether a model including FA values in the splenium of the CC at T1, age, and sex predicts verbal memory change. The null model, which included age and sex, accordingly, was not significant *R*^2^ = 0.009 (adjusted *R*^2^ = −0.023), *F*(2, 62) = 0.279, *p* = 0.757, Cohen’s *f* ^2^ = 0.01. The model including the FA values in the splenium of the CC at T1 was significant, *R*^2^ = 0.12 (adjusted *R*^2^ = 0.08), *F*(3, 61) = 2.79, *p* = 0.048, Cohen’s *f* ^2^ = 0.14, with the only significant predictor being the FA values in the splenium of the CC at T1, *β* = 0.34, *t* = 2.78, *p* = 0.007. Next, we added a group factor (participants enrolled in both music and language classes, participants enrolled in either music or language classes, participants enrolled in neither music or language classes) to test whether enrollment in music and/or language classes would improve the predictive power of this model. There was no significant *R*^2^ change, *p* = 0.158.

**Figure 3:**
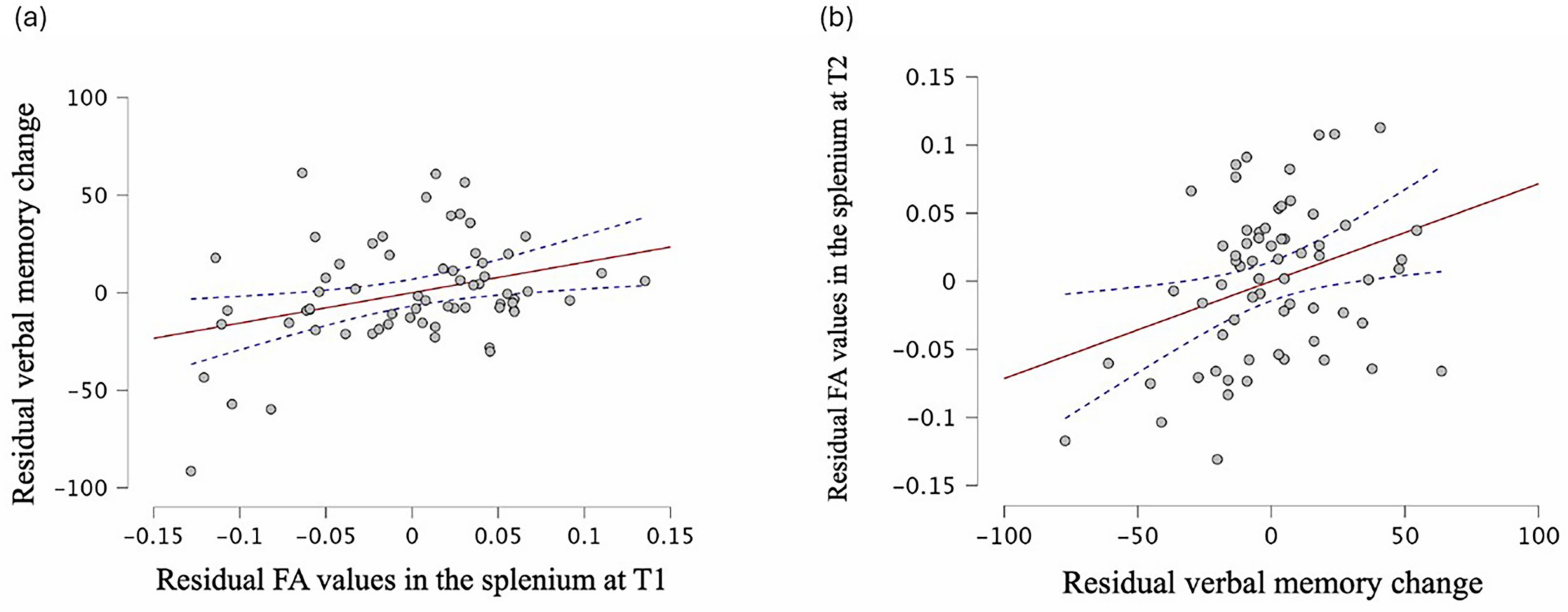
**(a)** Relationship between the residuals of FA values in the splenium at T1 and the residuals of verbal memory changes, with 95% confidence interval. **(b)** Relationship between the residuals of verbal memory changes and the residuals of FA values in the splenium at T2, with 95% confidence interval.

Another linear regression was conducted to examine whether verbal memory change can predict FA values in the splenium of the CC at T2. The null model, which included age and sex, accordingly, was not significant, *R*^2^ = 0.014 (adjusted *R*^2^ = −0.018), *F*(2, 62) = 0.443, *p* = 0.644, Cohen’s *f* ^2^ = 0.01. The model including verbal memory change significantly predicted FA values in the splenium of the CC, *R*^2^ = 0.126 (adjusted *R*^2^ = 0.083), *F*(3, 61) = 2.94, *p* = 0.040, Cohen’s *f* ^2^ = 0.14, with verbal memory change being the only significant predictor, *β* = 0.34, *t* = 2.80, *p* = 0.007. Adding the group factor (see above) did not lead to a significant *R*^2^ change, *p* = 0.788. Residuals are plotted in Figure 3.

Lastly, we assessed the effects of group, time, age, and sex on FA values in the splenium. None of the main or interaction effects were significant, *p*s *> 0*.05.

## 4 Discussion

Our analyses show that two different types of training can lead to significant improvements in verbal memory performance after one year. Our findings were specific to verbal memory, aligning with meta-analyses regarding effects of music training on verbal memory and a bilingual advantage for memory (Grundy and Timmer, 2017; Talamini et al., 2017). Additionally, our analyses showed that verbal memory performance change relates to white matter characteristics of the splenium of the CC. However, the shown training effects are not linked to this relationship between callosal white matter characteristics and verbal memory.

### 4.1 Training effects on verbal memory

In our first analysis, we examined the effects of music training on verbal and visuospatial memory scores. A significant change in verbal memory over time was observed within the group enrolled in the music classes but not in the group of participants who were not enrolled in music classes (see Figure 1a). In contrast, no significant changes were observed for visuospatial memory. Our results add longitudinal support for the conclusions of a recent meta-analysis based on mostly cross-sectional comparisons of musicians and non-musicians (Talamini et al., 2017). Our results extend the range of existing longitudinal evidence from studies involving children (Ho et al., 2003; Rickard et al., 2010; Roden et al., 2012), older adults (Galloway and Strong, 2021; Strong, 2022), and patient populations (Vik et al., 2018) to also include young adults.

In our next analysis, we examined the effects of language learning on verbal and visuospatial memory scores. Participants who enrolled in a language class showed significant improvements in verbal memory across two time points. Our results align with a meta-analysis comparing bilinguals and monolinguals in working memory capacity (Grundy and Timmer, 2017) as well as with more recent findings (Cockcroft et al., 2019; Fyndanis et al., 2023), linking language experience to enhanced verbal memory. However, unlike studies comparing bilinguals and monolinguals in a cross-sectional dataset, our participants actively participated in language learning and were assessed longitudinally; thus, improvements were associated with participation in language classes. While direct comparisons are limited, our findings support the idea that language learning activities benefit memory functions. This stands in contrast to conclusions reached by another longitudinal language learning study in which change in memory was negligible, though notably the studied duration of training was shorter and participants were older adults (Berggren et al., 2020).

We further found a significant linear contrast on verbal memory change such that participants who were enrolled in both music and language classes showed the greatest change in verbal memory performance, whereas participants enrolled in either showed a lesser degree of change (see 2c). Our results stand in contrast to conclusions reached from the analysis of cross-sectional datasets. In a sample of older adults, only the number of known foreign languages but not the hours per week spent playing instruments related to verbal memory performance (Fyndanis et al., 2023). In a sample of young adults, only musicianship but not bilingualism was associated with greater working memory performance (D’Souza et al., 2018; Moradzadeh et al., 2015). These conflicting results may have arisen due to differences in the used memory task. Additionally, one might argue that the hours spent playing instruments is only a vague estimation of the time spent in music training and that a more holistic assessment of music training may have led to different results in the study on older adults (Fyndanis et al., 2023).

There are two possible explanations for the compounded effect in our sample: The observed pattern could be due to the amount of time spent in relevant classes. Given that participants who were enrolled in both music and language classes were enrolled simply in more relevant classes, they might show a greater effect. Though we are not aware of studies with this direct comparison, results from cross-sectional studies suggesting a linear relationship between duration of music training and verbal memory (Ho et al., 2003; Roden et al., 2012; Strong and Midden, 2020) and suggestions of an age-of-acquisition dependent bilingual advantage in verbal memory would support this hypothesis (Delcenserie and Genesee, 2017).

Alternatively, the observed pattern is due to different contributions of music training and language learning on verbal memory, which however cannot be distinguished within the CVLT. Future studies may want to study the exact contributions of music training and language learning, for example, by studying whether there is a difference in the influence of the two types of experience on verbal processing which might contribute to performance on the CVLT (Allen and Hulme, 2006; Ferreri and Verga, 2016; Franklin et al., 2008; Smith and Geva, 2000). Nevertheless, to the best of our knowledge, we are the first to show the possibility of a compounded effect of music training and language learning on verbal memory, suggesting that engagement with both activities, for example, as part of training in singing or opera performance, could offer more robust transfer effects. To this effect, in a recent pre-print, researchers have reported increased callosal thickness in opera singers (Kleber et al., 2025).

Our additional analyses also confirmed that there was no significant relationship between changes in verbal memory percentile scores and prior music or multilingual proficiency measured by GoldMSI and LEAP-Q, respectively (*p*s *> 0*.05) Therefore, our results can be interpreted that the changes in verbal memory score found in the group of participants who received music training and/or language learning are not attributable to their previous experience, rather to the effects of the training they actively engage in. This might also explain the difference in findings between our study and those who have studied music training, language learning, and memory (D’Souza et al., 2018; Fyndanis et al., 2023; Moradzadeh et al., 2015), as there, memory performance was related to past experience.

Note, that there were no significant group differences at either time point, meaning that while music training and language learning led to improvements in verbal memory, they were not based on existing differences between groups nor did they lead to significant group differences. This result stands in contrast to previous studies that have reported group effects of musical and/or language expertise on memory performance, possibly due to differences in group sizes and the studied age group (Cockcroft et al., 2019; Fyndanis et al., 2023; Strong and Midden, 2020; Talamini et al., 2017). Moreover, in our study, the groups classified as” not enrolled in music training” or” not enrolled in language learning” within the first two analyses reported in section 3, the control participants in a manner of speaking, included participants with significant language experience and with significant musical experience respectively, which - as shown here - makes it less likely to find a group effect. Our findings thus suggest that not all control groups are equal and different pathways to improvements in verbal memory exist.

### 4.2 White matter characteristics and verbal memory change

In our analysis on the relationship between music training, language learning, memory, and the brain, we specifically focused on regions-of-interest which were determined based on available literature on the white matter tracts commonly associated with memory performance. First, we determined that among the four ROIs, only the splenium’s FA values significantly related to verbal memory change. Thus, we chose to focus our subsequent analyses only on the FA values in the splenium.

After controlling for the covariates of age and sex, FA values in the splenium of the CC at T1 were positively associated with changes in verbal memory performance (Figure 3a), and verbal memory change significantly predicted FA values in the splenium of the corpus callosum at T2 (Figure 3b). This implies that participants with greater memory improvement tended to have higher FA values in the splenium of the CC as did the previous literatures (Kennedy and Raz, 2009; Tang et al., 2010). A relationship between FA values in the CC and verbal memory performance has been previously reported in the populations with traumatic brain injury (Arenth et al., 2014; Yiannakkaras et al., 2019) and agenesis of the CC (Erickson et al., 2014). In the latter study, the researchers reported significantly lower scores in verbal memory test overall in participants with agenesis of the CC compared to a healthy control group. Notably, the only significant group difference was found on delayed memory tasks, which the authors interpreted as indicative of deficits in encoding rather than in retention or retrieval processes. However, contrary to what others have suggested (Douet and Chang, 2015; Fletcher et al., 2013), we did not observe a significant correlation between FA values in the fornix and memory performance.

Adding the group factor, that is, information regarding whether participants were enrolled in music and language classes, music or language classes, or neither, did not significantly change the explanatory power of the model, suggesting that the relationship of FA values in the splenium of the CC with changes in verbal memory is not mediated by participants’ type of training. Thus, while music training and language learning were related to verbal memory changes (see above), the improvements through enrollment in classes did not stem from an effect of music training and/or language learning on the white matter characteristics of the splenium. A possible explanation for this pattern of result is that music training and/or language learning influence a cognitive process, supported by another white matter tract, which influences performance on the CVLT.

One domain-general cognitive process which may likely play a role in CVLT performance is attention (Donders, 2008). Research has suggested effects of music training and language learning on attention (Bialystok and DePape, 2009; George and Coch, 2011). This cognitive process has been linked to white matter characteristics of the superior longitudinal fasciculus (Nakajima et al., 2020), and several studies have suggested that musicians and bilinguals, in contrast to non-musicians and monolinguals, show distinct white matter characteristics in this tract (Acer et al., 2018; Anderson et al., 2018; Kuhl et al., 2016; Luk et al., 2011; Mamiya et al., 2016; Oechslin et al., 2010; Pliatsikas et al., 2015). As suggested earlier, another process which may be influenced by training and relate to CVLT performance is verbal processing, which has been linked to white matter characteristics of the arcuate fasciculus (Nakajima et al., 2020). White matter characteristics of the arcuate fasciculus have also been shown to vary as a function of music training or language learning (Acer et al., 2018; Bengtsson et al., 2005; Cui et al., 2024; Halwani et al., 2011; Hämäläinen et al., 2017; Li et al., 2021; Loui et al., 2011a; Oechslin et al., 2010; Vaquero et al., 2018; Vaquero et al., 2020; Wei et al., 2024). Future research thus may want to more closely examine whether differences in white matter characteristics of the tracts subserving these other cognitive processes contributing to the performance on the CVLT can explain training effects on verbal memory.

### 4.3 Limitations

Due to recruitment issues, the groups of participants in our study are relatively small and uneven (as is unfortunately common in research on differences in brain structures in participants with specific training such as music training, e.g., group sizes range from *n* = 7 (Acer et al., 2018) to *n* = 18 (Steele et al., 2013) in recent studies with *n* = 59 (Jünemann et al., 2022) being a notable exception). This raises the risk of Type II errors for between group comparisons in particular. Thus, our conclusions regarding the absence of group differences should be treated with caution. Note also that our “control” participants in our analyses of the effect of music training and language learning on verbal memory performance included participants receiving language classes and music classes, respectively. This training may have also potentially diluted group contrasts.

Additionally, we were unable to control for the intensity or duration of training during the study. Unlike previous studies we reviewed, which compared bilinguals and multilinguals to monolinguals, participants in our study were enrolled in language training, which allowed us to assume that enrollment in classes might have led to respective training. Future studies may consider want to exert greater control over the intensity, duration, and contents of training to better elucidate cognitive mechanisms underlying improvements from training, so that dose–response effects can be inferred. Furthermore, as outlined earlier, we cannot separate the two possible explanations for the compounding effects nor isolate the specific contributions of music training and language learning on changes in verbal memory performance and thus remain uncertain of the direct causal relationships underlying these changes.

Future research may address these limitations and consider different ROIs, which have been shown to be influenced by both music training and language learning, such as the superior longitudinal fasciculus or the arcuate fasciculus. Given our focus on memory performance, here, we elected to only study ROIs which have been indicated in previous research to be linked to memory performance and also be implicated in group comparisons in music training or language learning studies. We believe investigation on other tracts, which are rarely the focus in studies on the neural basis of verbal memory, as well as the inclusion of measures of other relevant cognitive processes, could provide further insight into how music training and/or language learning contribute to verbal memory performance.

Another productive avenue for future research will be to investigate whether individual differences in verbal memory may relate to individual differences in absolute pitch. Absolute pitch refers to the ability to label a tone without a reference note, and is thus thought to involve multiple mnemonic processes (Van Hedger et al., 2024). While the ability to store the absolute pitch of different stimuli seems to be more common, the ability to explicitly label these stimuli is more rare (Frieler et al., 2013). Individual differences in pitch labeling ability are related to white matter characteristics of tracts connecting brain regions which are commonly involved in auditory processing, including the left planum temporale (Burkhard et al., 2020) and the left superior and middle temporal gyrus (Loui et al., 2011b). Given the possible relationship between verbal and musical memory (Williamson et al., 2010), future studies may also want to recruit additional participants with absolute pitch to specify the contributions of musical memory. One prediction would be, that a group of participants with absolute pitch who are receiving both musical training and engaging in language learning would show even greater improvements in verbal memory.

Lastly, it should be noted that there is ongoing debate about how to interpret FA values given their restricted reliability due to crossing fibers (Figley et al., 2022). However, here, we focused on FA as a white matter characteristic to make our findings more comparable to past research which has repeatedly linked this particular measure to our variables of interest and to reduce our risk of Type I errors. Further, the corpus callosum especially is known as a white matter tract with very few crossing fibers [see Figley et al. (2022)], allowing more reliable interpretation of FA values for this particular white matter tract.

## 5 Conclusion

In summary, our results show evidence that longitudinal music training and language learning lead to improvements in verbal memory. These improvements can also compound when participants engage in both music training and language learning, led to higher improvements between two time points. We found that verbal memory change is related to FA values in the splenium of the CC. Our findings partially align with the evidence from previous literature, in which musicians and bilinguals showed better memory performance and distinct white matter characteristics. However, there were no group differences or effects on these characteristics, suggesting that the contribution of music training and language learning to verbal memory may not be through improvement of memory processes subserved by the splenium of the CC but rather through improvement of other processes which may have an influence on the performance in verbal memory tasks.

## Data Availability

The raw data supporting the conclusions of this article will be made available by the authors, without undue reservation.
